# Classification of Regional Healthy Environment and Public Health in China

**DOI:** 10.3390/ijerph20053809

**Published:** 2023-02-21

**Authors:** Cheng Hu, Wulin Pan, Li Wen, Wei Pan

**Affiliations:** 1 School of Economic and Management, Wuhan University, Wuhan 430072, China; 2 School of Applied Economics, Renmin University of China, 54 Zhongguancun Street, Haiding District, Beijing 100872, China

**Keywords:** healthy environment, evaluation, classification, population health, environmental factors

## Abstract

Environmental pollution has become a hot topic of concern for the government, academia and the public. The evaluation of environmental health should not only relate to environmental quality and exposure channels but also the level of economic development, social environmental protection responsibility and public awareness. We put forward the concept of the “healthy environment” and introduced 27 environmental indicators to evaluate and classify the healthy environment of 31 provinces and cities in China. Seven common factors were extracted and divided into economic, medical, ecological and humanistic environment factors. Based on the four environmental factors, we classify the healthy environment into five categories—economic leading healthy environment, robust healthy environment, developmental healthy environment, economic and medical disadvantageous healthy environment and completely disadvantageous healthy environment. The population health differences among the five healthy environment categories show that economic environment plays a major role in population health. Public health in regions with sound economic environment is significantly better than that in other areas. Our classification result of healthy environment can provide scientific support for optimizing environmental countermeasures and realizing environmental protection.

## 1. Introduction

Climate change and other interdependent environmental disruptions caused by human activity have been recognized as the greatest threats ever faced by us [1]. The most direct and easily felt consequence of environmental pollution is the degradation of the quality of the human environment, affecting human physical health and production activities. For example, long-term exposure to PM_2.5_ might harm residential health and can increase premature death from specific diseases [2], as well as significantly increase the population’s risk of heart disease, stroke, chronic obstructive pulmonary disease and lung cancer [3,4]. Improvements in air quality are associated with greater life expectancy, reduced infant mortality, higher property values, increased productivity, higher earnings and other benefits [5]. Water pollution worsens the quality of the water environment and generally degrades the quality of drinking water sources, threatening people’s health and causing infertility and fetal malformations. In addition, exposure to bioaccumulated antibiotics poses serious health risks to ecosystems and humans [6].

This relationship between the environment and human health has been prominent amongst the concerns of international organizations including the World Health Organization (WHO), the World Bank, the United Nations, the Organization for Economic Co-operation and Development and the European Union (EU) [7]. Current research on how the environment affects residents’ health mainly falls into two categories. The first category is outdoor environment, which is divided into natural environment such as air quality, water pollution and social environment. For example, aquatic environments have deteriorated substantially due to the accumulation of toxic inorganic and organic pollutants that cause potentially adverse, widespread and detrimental effects on human health [8]. Jing, et al. [9] analyzed the change characteristics of PM 2.5 and O_3_ concentrations in 34 atmospheric environmental monitoring stations in Beijing from 2014 to 2020 and evaluated the health effects of air pollution prevention; results show that there were 790 excess deaths related to PM 2.5 and 2180 excess deaths related to O_3_ in 2019. In addition, more studies tend to discuss social environments, such as the community environment [10,11], residential environment [12], neighborhood environment and other outdoor factors on residents’ health. Neighborhood environments are considered crucial determinants of self-rated health [13]. Liu, et al. [14] showed that the residential environment is associated with older adults’ health directly, and also indirectly through a series of significant behavioral (physical and social activities) and perceptual (subjective well-being) factors.

The second category is how indoor environments impact on residents’ health. The influences of indoor environment quality on occupant health have long been one of the main focuses in built environment and public health research. Previous studies have identified various indicators for indoor environmental quality, including indoor air quality, thermal comfort, ventilation, visual condition, and acoustic condition [15]. Scholars are more inclined to directly discuss the impact of built environments on residents’ health [16]. The built environment affects public health behavior [17], public physiological health [18] and public mental health [19]. Tang, et al. [20] used exploratory factor analysis to identify a construct of housing/neighborhood factors and performed principal component regression to assess the relationship between the built environment and both self-rated physical health and mental health. In addition, much literature depicts a worldwide democratic advantage in population health [21].

The rapid deterioration of the ecological environment and the increasingly fierce social competition aggravate people’s internal and external pressures and increase the prevalence of occupational diseases and mental diseases. This phenomenon has also become a hot and important topic for government, academia and the public. Health policy analysts cannot overlook the importance of the environment to human health. The European Union funded the Integrating Environment and Health Research project (HERA) to set priorities for the future European research agenda in the environment, climate and health nexus [22]. In order to build a healthy environment, safeguard public health, strengthen environmental health management, and improve China’s ability to prevent and control environmental risks, the Chinese government has formulated the 14th Five-Year Plan for Environmental Health Work, proposing to strengthen monitoring and assessment of environmental health risks, vigorously improve residents’ environmental health literacy, and continue to explore countermeasures for environmental health management. In addition, it points out that environmental health standards should be further improved to achieve multi-level, diversified and distinctive development.

Aiming at the health problems brought by the environment, many studies have established indicators to comprehensively evaluate environmental health risks and assess the level of regional environmental health risks. Wu, et al. [23] constructed a lake ecological environment health assessment index system based on the driving-forces pressure-state-responses framework. Mapar, et al. [24] developed a performance evaluation tool with 80 indicators to monitor the health, safety, and environmental aspects of sustainable development. Zhang, et al. [25] established an indicator system that can reflect the comprehensive risks of environment and health, and divided the assessment result into four types according to risk level. Seventeen specific indicators cover social and economic development, pollution emission intensity, air pollution exposure, medical and public health, culture and education and so on. Furthermore, for human health indicators, scholars may draw on environmental exposure, human morbidity/mortality or well-being and sustainability approaches [26]. Canavese, et al. [27] presented a proposal for a system that can evaluate human health and urban environment sanitation in an integrated manner. The indicators selected for human health included child mortality rates due to acute diarrhea and acute respiratory tract infections.

We can find from the previous studies the following. First, scholars mainly discuss the impact of a certain type of environment, such as natural environment or indoor and outdoor environment on residents’ health, and few of them measure them from multiple aspects. Second, the existing indicators to measure residents’ health are more focused on the death rate or prevalence rate of certain diseases, lacking integrity and representation of the population. Third, the assessment concentrates on the level of regional environmental health risk, with assessment results of high or low risk, and fail to clarify the environmental risk characteristics through further segmentation. Generally, the environment includes a variety of environmental categories such as natural environment, cultural environment and economic environment, but the “healthy environment” is rarely put forward. What is a “healthy environment”? We define “healthy environment” as a healthy, safe and comfortable social environment that can secure for the public a good physical and mental state by meeting the public’s physiological, psychological and social demands. Therefore, the evaluation of a “healthy environment” should not only be linked to ecological factors but also be integrated into the overall evaluation system along with the social and economic environment, the medical environment and other aspects of human life. The study of the healthy environment index system provides theoretical guidance and a qualitative and quantitative basis for us to measure and judge healthy environments.

Based on various indicators such as the economic and social environment, we evaluated and classified the healthy environment of 31 provinces and cities in China, discussed the characteristics of different kinds of healthy environment, and discussed their relationship with public health. The main possible innovation in our work lies in the concept of the “healthy environment” and its classification. Through four types of environmental factor, we evaluated and classified regional healthy environments. We believe that this triage is really important because public health can behave differently in different types of health settings. For example, Mitchell [28] concludes that physical activity in natural environments is more associated with a reduction in the risk of poor mental health than in other environments; activity in different types of environment may promote different kinds of positive psychological responses.

## 2. Research Design

### 2.1. Variable Selection and Data Description

Indicators are measurements selected to represent a larger phenomenon of interest to the researcher [26], in our case, the relationships between population health and a healthy environment. However, the public’s living environment is not a self-contained, homogenous entity, but a complex system characterized by a number of different structures (e.g., educational, economic, mobility and political structures) that have their own dynamics and interact with each other in a complex grid [29]. Therefore, the index system of the healthy environment proposed consists of four parts—medical environment, economic environment, ecological environment and humanistic environment (Table 1).

The medical environment mainly reflects medical infrastructure and public health awareness, including the proportion of maternity insurance, the number of practicing (assistant) physicians per 1000 persons, the number of health institutions and so on. The economic environment not only takes basic economic indicators into account, but also includes consumption, expenditure and disposable income per capita to reflect residents’ income and consumption levels. The ecological environment includes measures of the energy intensity and emission intensity of polluting gases, which represent the environmental factors that may affect regional air quality and population health. In addition, urban environmental protection investment represents governments’ emphasis on environment, and the water resources and green park area per capita are used to represent the regional environmental quality. The humanistic environment covers educational attainment level, internet penetration rate and so on. Finally, 27 indicators were selected to build the whole healthy environment system (Table 1). The data cover 31 provinces and municipalities in China from 2010 to 2020, and mainly come from the China Statistical Yearbook, China Environmental Statistical Yearbook and China Health Statistical Yearbook.

### 2.2. Research Methods

#### 2.2.1. Factor Analysis

Factor analysis can be crudely described as an extension of the correlational method. When several variables are found to be rather highly correlated, it may be inferred that they are connected in some way, perhaps by a common underlying variable which is not immediately present in the measurements, but which nevertheless would account for them to a major extent [30]. Therefore, factor analysis is a method to find a few random variables that can synthesize the main information of all variables by studying the internal dependence of the correlation matrix among multiple variables. These random variables cannot be directly measured, so they are usually called common factors, and each common factor is irrelevant to the others. All variables can be expressed as a linear combination of common factors. The purpose of factor analysis is to reduce the number of variables and replace all variables with a few common factors. Equipped with n samples and p indicator, $X={\left(X_{1},X_{2},\ldots ,X_{p}\right)}^{T}$ is the random vector, and $F={\left(F_{1},F_{2},\ldots ,F_{m}\right)}^{T}$ is the common factor.

$$X_{1}=a_{11}F_{1}+a_{12}F_{2}+\ldots +a_{1m}F_{m}+\varepsilon_{1}$$

$$X_{2}=a_{21}F_{1}+a_{22}F_{2}+\ldots +a_{2m}F_{m}+\varepsilon_{2}$$

$$X_{p}=a_{p1}F_{1}+a_{p2}F_{2}+\ldots +a_{pm}F_{m}+\varepsilon_{p}$$

The above model is called the factor model. Matrix $A=\left(a_{ij}\right)$ is called the factor load matrix, $a_{ij}$ is the factor loading, and its essence is the correlation coefficient between common factor $F_{i}$ and vector $X_{j}$, which means how much variable $X_{j}$ depends on $F_{i}$. $\varepsilon_{j}$ is a special factor, representing the variable variation caused by the other unexpected influencing factors. The task of factor analysis is to analyze the internal correlation structure between variables. Larger samples outperform smaller samples due to the reduction in the probability of errors. Various recommendations pertaining to sample size can be found in the literature. While some authors highlight the importance of absolute sample size, most researchers focus on the ratio between subjects and variables and recommendations frequently include ratios of 5:1 or 10:1 [31]. In addition, the variables should be correlated. If variables are independent of each other, common factors cannot be extracted. This can be determined by Bartlett’s sphericity test and a correlation indicator, KMO test statistic, whose value is between 0 and 1. The closer the statistical value is to 1, the stronger the partial correlation between variables is, and the better the effect of factor analysis is. During the actual analysis process, when the KMO test statistic is greater than 0.7, factor analysis will generally present a better result.

The principal component method is often used to extract common factors. This method assumes that the variable is a linear combination of various common factors, to make the variance of the variable be explained by the principal component as much as possible, and ensure that the interpretation ratio of each common factor decreases successively. In the total variance interpretation table, common factors are extracted according to the default criterion of eigenvalues greater than 1. In addition, it should be emphasized that each common factor in factor analysis should have practical significance. To make the coefficient in the factor load matrix more significant, the initial load matrix can be rotated to redistribute the relationship between the common factor and the original variable, so that the absolute value of the correlation coefficient is differentiated to the two ends of the interval (0,1), and obtain more explicit results and make the interpretation of each common factor more meaningful. The commonly used factor rotation method is maximum variance orthogonal rotation (varimax), which maximizes the variance difference of common factors as far as possible to facilitate the interpretation of factors.

#### 2.2.2. K-Means Clustering Method

We use a non-hierarchical clustering method to divide the categories into 5 categories. The purpose of non-hierarchical clustering is to quickly divide cases into K categories. Generally, the specific number of categories needs to be determined before classification, and the entire analysis process is carried out iteratively. The K-means clustering steps are as follows:

1. Firstly, we determine the cluster number and divide the categories into 5 categories according to different environmental factors.
2. According to the specified clustering center, or the center of the structure of the data itself, we set the initial clustering center.
3. Next we calculate the distance between each case and the initial clustering center, classify them into each category according to the principle of minimum distance, and calculate the new clustering center of each category. Euclidean distance is commonly used to measure the distance between sample $X_{i}={\left(X_{i1},X_{i2},\ldots ,X_{ip}\right)}^{T}$ and $X_{i}={\left(X_{j1},X_{j2},\ldots ,X_{jp}\right)}^{T}$; the formula is:
  $$Euclid_{i,j}=\sqrt{{\left(X_{i1}-X_{j1}\right)}^{2}+{\left(X_{i2}-X_{j2}\right)}^{2}+\ldots +{\left(X_{ip}-X_{jp}\right)}^{2}}$$
4. According to the new clustering center, we recalculate the distance between each case and the new clustering center, and reclassify and update the category clustering center.
5. Step (4) is repeated until the moving distance of all clustering centers is less than 2% of the minimum moving distance of the initial clustering center, or the maximum number of iterations specified is reached.

## 3. Results

### 3.1. Factor Analysis Result

**Figure 1:**
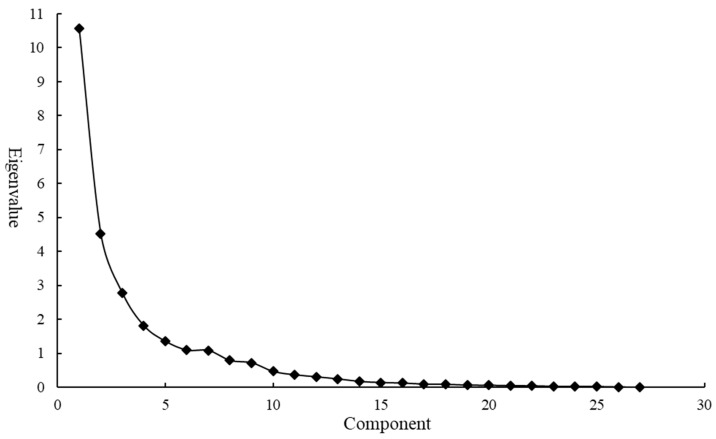
Lithotripsy diagram.

The exploratory factor analysis model was used, and Bartlett’s spherical test was significant (*p* < 0.01),and the KMO test statistical value is 0.828 (Table A1), meaning a better information overlap among variables. Table A2 shows the variance of the common factor, and the information extraction proportion of most variables is above 80%, indicating that the proposed common factor has a strong explanatory ability for most variables. The common factor was extracted according to the default standard with an eigenvalue greater than one. Combined with the lithotripsy diagram (Figure 1), which is used to show the importance of each common factor, seven common factors are finally extracted. The horizontal axis of the lithotripsy diagram is the number of common factors, and the vertical axis is the eigenvalues which are arranged according to the eigenvalue order. The cumulative variance contribution rate of seven common factors was 85.909% (Table A3). After rotation, the information was redistributed with an unchanged cumulative variance contribution rate. The variance contribution rate of each of the seven common factors changed, and the gap between them decreased. The component matrix after rotation is shown in Table 2.

In the rotated component matrix, each variable is sorted according to the coefficient, and the absolute value of a coefficient less than 0.3 will not be output. Combining the results in Table 2 and the indicator classification in Table 1, we try to classify each factor into one that can reflect the economic environment, medical environment, ecological environment and humanistic environment. It can be seen that the first common factor $F_{1}$ has a large load in reflecting the overall economic situation, such as X13 household consumption expenditure per capita, X14 disposable income per capita and X11 GDP per capita, so common factor $F_{1}$ can be named as the economic environment factor. The second common factor $F_{2}$ has a large load in social, medical and health infrastructure, including X5 the number of health institutions and X6 community-level medical institutions, so we name $F_{2}$ as the medical environment factor. $F_{3}$ to $F_{6}$ have a large load on the indicators reflecting production and ecological investment, such as X20 water resources per capita, X15 energy intensity and X17 NO emission intensity, so they are named as ecological environmental factors. $F_{7}$ mainly includes population density and is named the humanistic environment factor. Each variable can be expressed as:

$$ZX_{1}=-0.334F_{1}+0.667F_{3}+0.436F_{4}+\varepsilon_{1}$$

$$ZX_{2}=0.627F_{1}-0.388F_{2}+0.408F_{5}+\varepsilon_{2}$$

……

$$ZX_{27}=0.813F_{1}-0.343F_{4}+\varepsilon_{27}$$

It should be pointed out that the variables above are normalized variables. The above functions represent each variable as a linear combination of common factors, but it is necessary to express the common factor as a linear form of each variable, which is also called the factor score function. Usually, the regression method is adopted to estimate the factor score. The essence of the regression method is to establish a regression equation between the original variable and the common factor. The component score coefficient matrix is shown in Table A4. The expression of each common factor is:

$$F_{1}=-0.019ZX_{1}+0.073ZX_{2}+\ldots +0.059ZX_{27}$$

$$F_{2}=0.011ZX_{1}+0.043ZX_{2}+\ldots -0.007ZX_{27}$$

……

$$F_{7}=-0.037ZX_{1}+0.104ZX_{2}+\ldots +0.026ZX_{27}$$

Through the above function, the environment score of seven common factors can be calculated. Since the above seven common factors reflect the assessment level of local healthy environments in different aspects, we take the proportion of the variance contribution rate (in Table A3) corresponding to each common factor as the weight to calculate the comprehensive score, namely:

$$Score=\frac{34.977}{85.909}F_{1}+\frac{13.055}{85.909}F_{2}+\frac{10.200}{85.909}F_{3}+\frac{8.857}{85.909}F_{4}+\frac{7.743}{85.909}F_{5}+\frac{6.216}{85.909}F_{6}+\frac{4.860}{85.909}F_{7}$$

### 3.2. Cluster Analysis Results

Based on the common factor score, we gain four environmental factor scores, economic environment factor score $S_{Economic}$, medical environment factor score $S_{Medical}$, ecological environment factor score $S_{Ecological}$, humanistic environment factor score $S_{Humanistic}$ and total score S. In order to explore the characteristics of different categories of healthy environment, we classified the scores of four environmental factors through the K-means clustering method. Based on the distance between each case after classification and the clustering center and combining the practical meaning of each category, we finally divide samples into five categories. The initial clustering center is shown in Table A5, the essence of which is the score of each environmental factor of a certain five cases in the sample.

**Figure 2:**
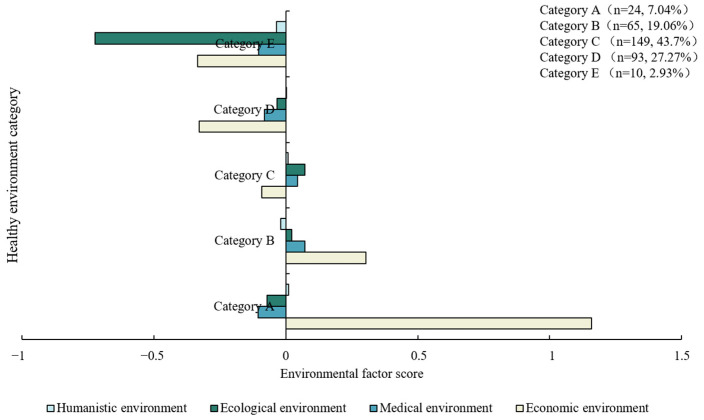
Classification characteristics of five healthy environments.

Table A6 show the iterative process records and the change of cluster center is obviously smaller until it finally approaches zero. We set the convergence criterion as 0.02. The iteration stops when the full iteration is unable to move any cluster centers by 2% of the minimum distance between any initial cluster centers. The whole iteration process is terminated at the seventh step, achieving convergence. The final clustering center is shown in Table 3, the essence of which is the average value of the score in each category, and can be used to describe the characteristics of five different types of healthy environment in the four environmental factors. Figure 2 shows the distribution of environmental factors in each healthy environment category. Category C has the largest number of cases, accounting for 43.7%, while Category E has the smallest number. Sometimes the number of classified cases can play an auxiliary role in determining the final category characteristics.

The most obvious weakness of cluster analysis is that cluster analysis can always obtain several types of results regardless of whether there are different categories in the data. Therefore, it is very important to verify the validity of clustering results. The ANOVA result in Table A7 shows that the environment factors scores are statistically different in different healthy environments.

**Figure 3:**
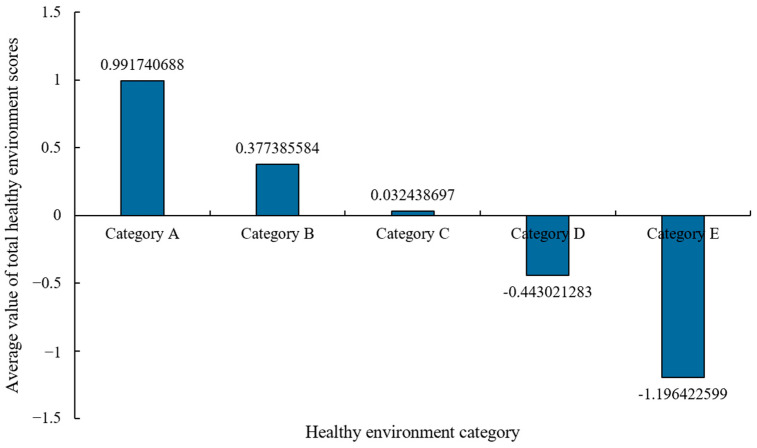
Average score of five healthy environment categories.

**Figure 4:**
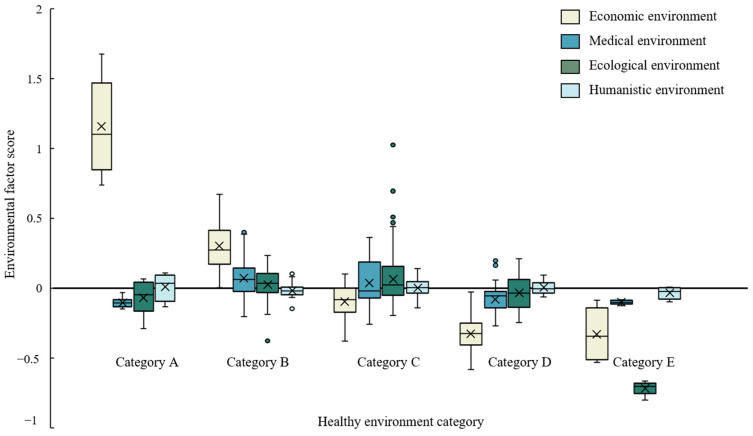
Distribution of four environmental factors in five healthy environments.

**Figure A1:**
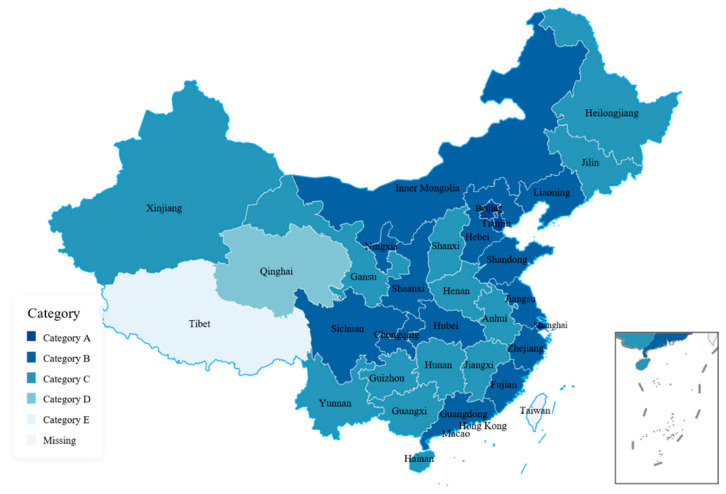
Regional healthy environment categories in 2020.

Figure 3 shows the mean scores of five healthy environment categories. The distribution of four environmental factor scores in various categories is shown in Figure 4. Table A8 and Figure A1 present the environmental factor scores and classification results of each region in 2020. In terms of the four environmental factor scores, the five healthy environments are differentiated. Category A is significantly ahead of other regions in economy, followed by Category B, while the others are relatively backward in economic environment, especially Category D and Category E, with significant economic disadvantages. None of them scored well in the medical environment factor. Relatively speaking, Category B has greater advantage in the medical environment, followed by Category C. Category A, D and E are lagging behind in the medical environment. In terms of ecological environment factors, Category B, C and D have comparative advantages, while the other areas have obvious disadvantages, especially Category E, which show significant ecological disadvantages. In the humanistic environment factor, all regions scored around 0. Category B and Category E have relative disadvantages.

The heterogeneity of cities in different healthy environment categories indicates that the healthy environment in different regions has its special development rules, and presents various characteristics. Category A is economic leading healthy environment, with strong economic advantages, and its economic environment score is much higher than other areas. Although it has certain disadvantages in medical environment and ecological environment, its superior economic environment still makes its average total score far higher than other categories, with an average value of 0.9917. Category B is a robust healthy environment with a total score of 0.3774, lower than Category A. A robust healthy environment has great comparative advantages in four environmental factors, especially in economic advantages. Despite the disadvantage in the humanistic environment, it is the most stable type of healthy environment. Category C is a developmental healthy environment with a mean total score of 0.0324, showing a weak comparative advantage. A developmental healthy environment has light comparative advantages in medical, ecological and humanistic environments. It has significant economic disadvantages and still needs to be further improved economically. Compared with the robust healthy environment, the developmental healthy environment lags behind economically. Category D is the economic and medical disadvantageous healthy environment, and the average score is −0.4430. Although it has obvious economic and medical disadvantages, it has certain ecological and humanistic advantages. Category E is a completely disadvantageous healthy environment and has the lowest mean score of −1.1964, with four negative environmental factor scores, and it has absolute ecological and economic environment disadvantages. Meanwhile, the disadvantages of the medical environment and the humanistic environment are also significant.

### 3.3. Population Health and Healthy Environments

When selecting diseases that represent population health, we select maternal mortality rate (1/100,000) and the mortality rates of Class A and Class B notifiable infectious diseases (1/100,000) rather than typical environment-related diseases such as lung cancer and bronchitis, to study the impact of healthy environment types on public health. Table 4 presents the descriptive statistics of the two diseases under different healthy environment types. Due to the unevenness of the classification results, a large variance difference exists, and the sample did not necessarily meet the homogeneity of variance. Therefore, the Games–Howell method based on variance inequality was used for the comparison between different categories.

The Games–Howell multiple comparison results are shown in Table 5. At the 5% significance level, there were significant differences in maternal mortality among different healthy environment categories, mainly reflected in that maternal mortality increased significantly with the decrease in the total healthy environment score. Take the robust healthy environment (Category B) as an example; its maternal mortality rate is 2.683/100,000 higher than that in economic leading healthy environments (Category A), and 6.504/100,000, 11.691/100,000 and 107.388/100,000 lower than the other three types of healthy environment, respectively. Similarly, at the 5% significance level, the mortality rate of Class A and Class B notifiable infectious diseases was significantly different among the five healthy environment categories. Specifically, the mortality rate of Class A and Class B notifiable infectious diseases in the economic leading healthy environment (Category A) is 0.5822/100,000 and 1.3801/100,000 lower than that in Category C and Category D, respectively.

## 4. Discussion

The five categories of healthy environment are economic leading healthy environment, robust healthy environment, developmental healthy environment, economic and medical disadvantageous healthy environment and completely disadvantageous healthy environment. There is a significant imbalance in regional economic development; only a few regions have a large economic advantage. All regions perform well in ecological environment, but the medical and humanistic environment needs to be improved.

The economic leading healthy environments include Beijing, Shanghai and Tianjin. These regions have significant advantages in the economic environment and complete disadvantages in the medical environment, and also have certain disadvantages in ecological and humanistic environments. Health indicators in these regions perform better than in other regions. It should be noted that due to the limitation of indicators, we only introduce basic medical facilities as the medical environment, and fail to take indicators that reflect local rich medical resources, such as medical equipment and doctors’ qualifications into account. We still insist that the results of our work are reasonable, since rich medical resources attract more patients, which means higher medical pressure and will squeeze on the medical care of local residents.

Robust healthy environments include Guangdong, Zhejiang, Jiangsu and so on, which have relative economic advantages and no obvious disadvantages in medical, ecological and humanistic environments. The maternal mortality rate in robust healthy environments was slightly higher than that in the economic leading environments and there was no significant difference in the mortality rate of Class A and Class B notifiable infectious diseases. Developmental healthy environments are mainly concentrated in Henan, Anhui and so on, with an economic disadvantage, and dominant in medical, ecological and humanistic environments. The maternal mortality rate was significantly higher than that in the economic leading healthy environments and robust healthy environments, and there was little difference in the mortality rate of Category A and Category B notifiable infectious diseases. The completely disadvantageous healthy environment is mainly in Xinjiang, where the health condition is poor and the mortality rate of diseases is significantly higher than that of other regions. Due to the small sample size, we will not elaborate it to avoid errors.

Based on the population health differences among the five healthy environment categories, we believe that the economic environment plays a major role in population health. Public health in areas with a good economic environment is significantly better than that in other areas. A better medical environment and ecological environment can also bring positive effects on population health. To further improve healthy environment levels in China, on the one hand, we should focus on enhancing the economic environment and reducing the gap between regional economies. On the other hand, it is necessary to improve the ecological and humanistic environment to enhance the social environment, so as to improve public health levels.

**Figure A2:**
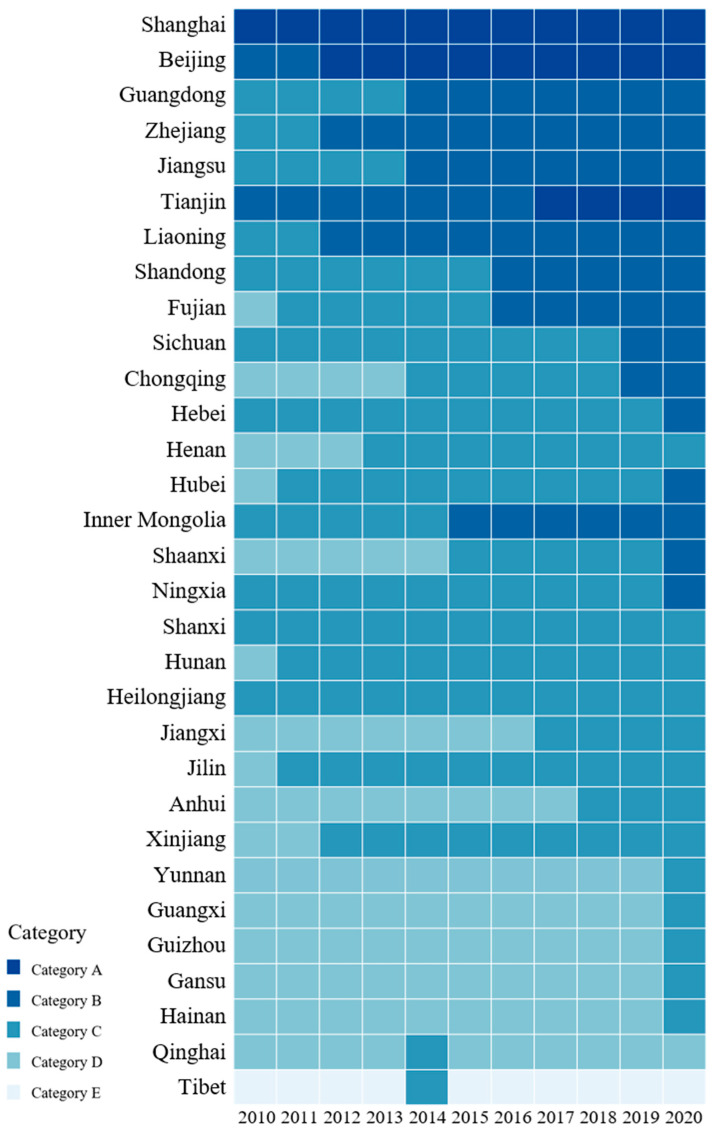
Historical trend of healthy environment categories in different regions.

Additionally, as can be seen in Figure A2, the regional healthy environment category has gradually developed in a better direction. Shanghai and Beijing have been economic leading healthy environments for a long time, while Qinghai and Tibet have shown greater weakness and basically remain in the same healthy environment category. Regional heterogeneity leads to different characteristics of health environment category changes in various regions. However, overall, economic factors play a significant role. Although economic development may bring about some health problems, our results suggest that higher economic levels still have a positive effect on population health. On the one hand, economic development will bring resources flow, including human and material resources and so on. This flow can create a development environment for the improvement of population health. On the other hand, residents in areas with higher economic levels generally have higher health literacy. They not only pay more attention to their health than people in economically disadvantaged areas, but also have a stronger ability and willingness to pay.

## 5. Conclusions and Policy Implications

The health environment evaluation itself should integrate theories of geography, ecology, climatology, social economics and other fields to guide the evaluation process. We put forward the concept of the “healthy environment” and divide 27 environmental indicators into economic environment, medical environment, ecological environment and humanistic environment to evaluate and classify the healthy environments of 31 provinces and cities in China. Based on the rotated principal component analysis matrix, seven common factors were divided into economic environment factors, medical environment factors, ecological environment factors and humanistic environment factors, to entitle factors to a practical meaning, and obtain scores of four environmental factors in each region. We classify the healthy environment into five categories based on the four environmental factor scores. Finally, the maternal mortality rate and the mortality rate of Class A and Class B notifiable infectious diseases are taken as population health indicators to discuss the population health differences under five healthy environments. In the context that current research is generally focused on the level of environmental health risk, it is meaningful to classify the healthy environment and figure out its characteristics. The overall health environment score in five healthy environments decreased successively, and public health also decreased gradually. Different healthy environment categories show different characteristics. Based on different environmental characteristics, we believe that the economic environment plays a major role in population health.

A good interactive relationship can be established between environment and health, and the understanding of this relationship will help decision-makers to have an insight into the possible consequences of policy implementation. The evaluation method of the healthy environment can provide scientific support for optimizing environmental countermeasures and realizing environmental protection and sustainable development of the economy and society. There are some deficiencies in selecting environment and health indicators. On the one hand, due to the availability of data, we can only take provinces and cities as the evaluation unit. On the other hand, the lack of detailed ecological environmental data such as air quality in the evaluation index may affect the accuracy of the evaluation results. Although there are some imperfections, it can still provide a new idea for the study of environmental health by constructing an indicator system and classifying the healthy environment.

## Figures and Tables

**Table 1:** Indicator system of healthy environment (N = 341).

Category	Code	Variable Description	Units	Min	Max	Mean	SD
Medical environment	X1	The proportion of personal health expenditure to total health expenditure	%	5.16	45.70	30.21	7.34
	X2	Health check-up proportion	%	0.03	0.29	0.12	0.05
	X3	Proportion of maternity insurance	%	0.02	1.23	0.26	0.18
	X4	Number of practicing (assistant) physicians per 1000 persons	Unit	1.04	5.851	2.34	0.66
	X5	Number of health institutions	Unit	4129.00	86,939.00	31,609.82	22,124.75
	X6	Number of community-level medical institutions	Unit	3878.00	83,972.00	29,951.35	21,308.79
	X7	Number of hospital beds	Unit	0.84	66.72	22.62	15.08
Economic environment	X8	Imports and exports as a percentage of GDP	%	0.01	2.06	0.30	0.35
	X9	The level of urbanization	%	0.23	0.94	0.57	0.13
	X10	Number of industrial enterprises above the scale	Unit	56.00	64,364.00	12,199.58	13,687.72
	X11	GDP per capita	CNY	12,882.00	164,158.00	53,394.11	27,145.92
	X12	Unemployment rate	%	1.20	4.60	3.28	0.64
	X13	Household consumption expenditure per capita	CNY	4809.00	45,605.00	15,876.74	7231.63
	X14	Disposable income per capita	CNY	6628.00	72,232.00	22,221.18	11,210.83
Ecological environment	X15	Energy intensity	Tons per billion CNY	0.21	18.59	1.27	2.19
	X16	SO_2_ emission intensity	Tons per billion CNY	0.05	254.26	33.94	41.38
	X17	NO emission intensity	Tons per billion CNY	2.41	291.51	39.71	37.58
	X18	Proportion of environmental pollution investment to GDP	%	0.04	30.37	1.61	1.95
	X19	Proportion of urban environmental infrastructure investment to GDP	%	0.00	21.66	0.98	1.33
	X20	Water consumption per capita	Cubic meter per capita	0.12	151.27	9.38	24.92
	X21	Green park area per capita	Square meter per capita	5.78	21.05	12.77	2.87
Humanistic environment	X22	Population density	People per square kilometer	515.00	5821.00	2839.06	1167.65
	X23	Number of minimum subsistence allowance in urban residents	Ten thousand people	2.50	189.30	52.06	41.60
	X24	The average schooling of people	Year	4.22	12.78	8.97	1.13
	X25	Proportion of people with higher education attainment	%	0.02	0.50	0.14	0.07
	X26	Library collection per capita	Volume per capita	0.18	3.25	0.69	0.51
	X27	Internet penetration rate	%	19.80	85.26	50.70	13.96

**Table 2:** Rotated Component Matrix.

	Component						
	$F_{1}$	$F_{2}$	$F_{3}$	$F_{4}$	$F_{5}$	$F_{6}$	$F_{7}$
X13	0.950						
X14	0.939						
X11	0.928						
X3	0.912						
X9	0.888		0.314				
X25	0.880						
X4	0.853						
X26	0.843						
X27	0.813			−0.343			
X24	0.732		0.591				
X8	0.714						
X2	0.627		−0.388			0.408	
X23	−0.425	0.343		0.350		−0.317	0.342
X5		0.935					
X6		0.932					
X7		0.899					
X10		0.624				0.569	
X20			−0.881				
X15			−0.806				
X1	−0.334		0.667	0.436			
X12				0.753			0.373
X17	−0.394			0.719			
X16	−0.392			0.689			
X19					0.975		
X18					0.955		
X21						0.904	
X22							0.830

**Table 3:** Final clustering center.

	Category A	Category B	Category C	Category D	Category E
Economic environment factor	1.16	0.30	−0.09	−0.33	−0.33
Medical environment factor	−0.11	0.07	0.04	−0.08	−0.10
Ecological environment factor	−0.07	0.02	0.07	−0.03	−0.72
Humanistic environment factor	0.01	−0.02	0.01	0.00	−0.04

**Table 4:** Descriptive statistics of two diseases.

Dependent Variable		Mean	SD	95% Confidence Interval	
				Lower Bound	Upper Bound
Maternal mortality rate	Category A	5.929	2.459	1.091	10.767
	Category B	8.612	1.494	5.673	11.552
	Category C	15.116	0.987	13.174	17.058
	Category D	20.303	1.249	17.846	22.761
	Category E	116.000	3.810	108.505	123.495
Mortality rate of Class A and B notifiable infectious diseases	Category A	0.628	0.299	0.040	1.216
	Category B	0.973	0.182	0.616	1.330
	Category C	1.211	0.120	0.975	1.447
	Category D	2.008	0.152	1.710	2.307
	Category E	0.807	0.463	−0.104	1.718

**Table 5:** Games–Howell multiple comparison results.

Variable	I	J	Mean Difference (I-J)	SD	*p*	95% Confidence Level	
						Lower Bound	Upper Bound
Maternal mortality rate (1/100,000)	Category A	Category B	−2.683 *	0.7014	0.003	−4.674	−0.692
		Category C	−9.187 *	1.0315	0.000	−12.039	−6.335
		Category D	−14.374 *	1.0987	0.000	−17.421	−11.327
		Category E	−110.071 *	16.4611	0.001	−165.393	−54.748
	Category B	Category C	−6.504 *	0.9426	0.000	−9.099	−3.909
		Category D	−11.691 *	1.0157	0.000	−14.503	−8.879
		Category E	−107.388 *	16.4558	0.001	−162.708	−52.068
	Category C	Category D	−5.187 *	1.2664	0.001	−8.671	−1.703
		Category E	−100.884 *	16.4732	0.001	−156.212	−45.556
	Category D	Category E	−95.697 *	16.4775	0.002	−151.027	−40.366
Mortality rate of category A and B notifiable infectious diseases	Category A	Category B	−0.3447	0.17430	0.287	−0.8325	0.1430
		Category C	−0.5822 *	0.11414	0.000	−0.8969	−0.2675
		Category D	−1.3801 *	0.20807	0.000	−1.9581	−0.8020
		Category E	−0.1787	0.10755	0.489	−0.5187	0.1613
	Category B	Category C	−0.2375	0.19910	0.756	−0.7892	0.3143
		Category D	−1.0353 *	0.26440	0.001	−1.7650	−0.3056
		Category E	0.1661	0.19540	0.914	−0.3826	0.7148
	Category C	Category D	−0.7979 *	0.22925	0.006	−1.4313	−0.1644
		Category E	0.4035	0.14432	0.058	−0.0094	0.8165
	Category D	Category E	1.2014 *	0.22604	0.000	0.5721	1.8307

* The mean difference was significant by 5%.

## Data Availability

All the data and software files are available at: Cheng, Hu (2023): environmental healthy data.xlsx. figshare. Dataset. https://doi.org/10.6084/m9.figshare.22126856.v1 (accessed on 13 February 2023).

## Declarations

Ethics approval: Not applicable; Consent to participate: Not applicable; Consent for publication: Not applicable.

## Appendix A

**Table A1:** KMO and Bartlett’s sphericity test.

KMO		0.828
Bartlett’s sphericity test	Approximate chi square	15,121.7
	df	351
	p	0

**Table A2:** Common factor variance.

	Initial	Extraction
X1	1.000	0.831
X2	1.000	0.769
X3	1.000	0.890
X4	1.000	0.850
X5	1.000	0.946
X6	1.000	0.942
X7	1.000	0.908
X8	1.000	0.598
X9	1.000	0.911
X10	1.000	0.852
X11	1.000	0.901
X12	1.000	0.772
X13	1.000	0.965
X14	1.000	0.946
X15	1.000	0.862
X16	1.000	0.797
X17	1.000	0.850
X18	1.000	0.966
X19	1.000	0.965
X20	1.000	0.870
X21	1.000	0.854
X22	1.000	0.748
X23	1.000	0.710
X24	1.000	0.916
X25	1.000	0.900
X26	1.000	0.834
X27	1.000	0.844

**Table A3:** Total variance explanation of common factors.

	Initial Eigenvalues			Extraction Sums of Squared Loadings			Sum of the Squared Loads after Rotation		
	Total	Percentage of Explained Variance	Cumulative %	Total	Percentage of Explained Variance	Cumulative %	Total	Percentage of Explained Variance	Cumulative %
1	10.553	39.086	39.086	10.553	39.086	39.086	9.444	34.977	34.977
2	4.525	16.759	55.845	4.525	16.759	55.845	3.525	13.055	48.032
3	2.776	10.280	66.125	2.776	10.280	66.125	2.754	10.200	58.232
4	1.812	6.712	72.837	1.812	6.712	72.837	2.391	8.857	67.089
5	1.354	5.015	77.852	1.354	5.015	77.852	2.091	7.743	74.832
6	1.093	4.047	81.899	1.093	4.047	81.899	1.678	6.216	81.048
7	1.083	4.010	85.909	1.083	4.010	85.909	1.312	4.860	85.909
8	0.789	2.924	88.833						
9	0.720	2.666	91.499						
10	0.466	1.724	93.223						
11	0.367	1.358	94.581						
12	0.309	1.144	95.724						
13	0.247	0.913	96.637						
14	0.172	0.637	97.275						
15	0.139	0.513	97.788						
16	0.123	0.456	98.244						
17	0.093	0.344	98.588						
18	0.087	0.323	98.911						
19	0.063	0.234	99.145						
20	0.058	0.216	99.361						
21	0.047	0.173	99.534						
22	0.042	0.157	99.690						
23	0.027	0.100	99.790						
24	0.027	0.099	99.889						
25	0.021	0.079	99.968						
26	0.008	0.031	99.999						
27	0.000	0.001	100.000						

**Table A4:** Coefficient matrix of component score.

	$F_{1}$	$F_{2}$	$F_{3}$	$F_{4}$	$F_{5}$	$F_{6}$	$F_{7}$
X1	−0.019	0.011	0.225	0.141	−0.023	0.049	−0.037
X2	0.073	0.043	−0.166	0.050	0.067	0.194	0.104
X3	0.119	0.028	0.001	0.065	0.017	−0.056	−0.122
X4	0.108	0.017	−0.081	−0.034	0.041	−0.111	0.084
X5	0.036	0.321	−0.071	0.037	0.051	−0.133	−0.035
X6	0.037	0.322	−0.073	0.039	0.051	−0.139	−0.039
X7	0.023	0.271	−0.040	−0.001	0.034	0.043	0.012
X8	0.094	0.039	0.002	0.094	−0.036	−0.013	−0.197
X9	0.103	−0.018	0.095	0.064	−0.014	0.000	−0.023
X10	0.001	0.163	0.023	0.090	−0.020	0.310	−0.235
X11	0.127	0.056	−0.039	0.075	0.017	−0.018	−0.023
X12	0.139	0.117	−0.208	0.489	−0.033	−0.002	0.299
X13	0.119	0.035	−0.032	0.037	0.015	−0.011	0.063
X14	0.121	0.051	−0.046	0.038	0.020	−0.016	0.053
X15	0.049	0.067	−0.339	0.180	0.047	−0.031	−0.060
X16	0.016	−0.061	0.065	0.313	0.003	0.060	−0.180
X17	0.030	−0.045	0.007	0.351	0.028	0.068	−0.172
X18	0.029	0.042	0.006	0.015	0.487	0.029	0.055
X19	0.026	0.062	0.043	−0.048	0.512	−0.026	0.052
X20	0.014	0.049	−0.368	0.019	−0.118	−0.112	0.006
X21	−0.074	−0.105	0.079	0.024	0.006	0.653	0.128
X22	−0.013	−0.078	0.028	−0.036	0.039	0.115	0.670
X23	0.029	0.112	−0.004	0.128	0.031	−0.163	0.209
X24	0.069	−0.038	0.218	−0.028	−0.007	−0.101	−0.032
X25	0.116	−0.010	0.034	−0.010	0.024	−0.205	−0.043
X26	0.160	−0.010	−0.115	0.260	−0.010	0.044	0.163
X27	0.059	−0.007	0.005	−0.065	0.018	0.088	0.026

**Table A5:** Initial clustering center.

Environment Factor	Cluster				
	1	2	3	4	5
Economic environment factor	1.67	0.67	−0.17	−0.32	−0.54
Medical environment factor	−0.07	0.12	0.09	0.22	−0.12
Ecological environment factor	0.07	0.15	1.04	0.03	−0.81
Humanistic environment factor	0.10	−0.03	0.06	0.07	−0.07

**Table A6:** Iteration history records.

Iteration	Changes in the Clustering Center				
	1	2	3	4	5
1	0.223	0.227	0.163	0.285	0.202
2	0.308	0.283	0.349	0.048	0.018
3	0.050	0.077	0.197	0.036	0.002
4	0.002	0.059	0.276	0.041	0.000
5	0.019	0.075	0.140	0.095	1.377 × 10^−5^
6	0.019	0.036	0.041	0.039	1.252 × 10^−6^
7	0.001	0.001	0.000	0.000	1.138 × 10^−7^

**Table A7:** Analysis of variance.

	Cluster		Error		F	*p*
	Mean Square	df	Mean Square	df		
Economic environment factor	12.555	4	0.022	336	564.323	0.000
Medical environment factor	0.322	4	0.020	336	16.045	0.000
Ecological environment factor	1.411	4	0.023	336	62.261	0.000
Humanistic environment factor	0.015	4	0.003	336	4.709	0.001

**Table A8:** Healthy environment scores and classification in 2020.

Area	Economic Environment	Medical Environment	Ecological Environment	Humanistic Environment	Total Score	Category
Shanghai	1.6745	−0.0725	0.0671	0.1038	1.772921	A
Beijing	1.618	−0.0329	−0.2581	−0.0661	1.260983	A
Guangdong	0.5761	0.2641	0.1291	−0.0101	0.959204	B
Zhejiang	0.6736	0.1175	0.1513	−0.0302	0.912177	B
Jiangsu	0.5993	0.1855	0.0974	−0.0309	0.851311	B
Tianjin	0.8008	−0.1466	−0.0138	0.0801	0.720555	A
Liaoning	0.426	0.0697	0.033	0.0169	0.54558	B
Shandong	0.2942	0.4087	−0.1196	−0.0634	0.51985	B
Fujian	0.4319	0.0077	−0.0022	0.0565	0.493836	B
Sichuan	0.1743	0.385	−0.1723	0.0357	0.422662	B
Chongqing	0.3315	0.0016	−0.0351	0.0345	0.332549	B
Hebei	0.1276	0.331	−0.1461	0.002	0.314606	B
Henan	−0.0445	0.285	−0.0155	0.0762	0.301236	C
Hubei	0.1795	0.0793	−0.0134	0.0341	0.279583	B
Inner Mongolia	0.279	−0.0309	−0.0286	0.0073	0.22675	B
Shaanxi	0.1466	−0.0024	−0.0278	0.1027	0.21904	B
Ningxia	0.1709	−0.2074	0.1455	0.0796	0.18863	B
Shanxi	0.0281	−0.0175	0.0556	0.0564	0.122577	C
Hunan	−0.0141	0.1686	−0.0783	0.0299	0.106175	C
Heilongjiang	0.019	−0.0909	0.0099	0.122	0.060059	C
Jiangxi	−0.0294	0.0152	−0.0431	0.0709	0.013597	C
Jilin	0.0755	−0.0668	−0.0431	0.0047	−0.02955	C
Anhui	−0.0742	0.0204	−0.0038	0.0021	−0.05552	C
Xinjiang	0.025	−0.1182	−0.0396	0.0465	−0.08619	C
Yunnan	−0.074	0.0044	−0.1482	0.0541	−0.16375	C
Guangxi	−0.096	0.015	−0.1003	−0.0097	−0.19103	C
Guizhou	−0.0816	0.0121	−0.1741	0.0332	−0.2104	C
Gansu	−0.1318	−0.0773	−0.0683	0.0461	−0.23137	C
Hainan	0.0464	−0.1983	−0.1904	0.0028	−0.33956	C
Qinghai	−0.0495	−0.2209	−0.2497	0.0132	−0.50688	D
Tibet	−0.0888	−0.117	−0.6768	−0.0029	−0.88541	E
